# Obesity-promoting and anti-thermogenic effects of neutrophil gelatinase-associated lipocalin in mice

**DOI:** 10.1038/s41598-017-15825-4

**Published:** 2017-11-14

**Authors:** Akira Ishii, Goro Katsuura, Hirotaka Imamaki, Hiroyuki Kimura, Keita P. Mori, Takashige Kuwabara, Masato Kasahara, Hideki Yokoi, Kousaku Ohinata, Tomoko Kawanishi, Junichi Tsuchida, Yuji Nakamoto, Kazuwa Nakao, Motoko Yanagita, Masashi Mukoyama, Kiyoshi Mori

**Affiliations:** 1 Department of Nephrology, Graduate School of Medicine, Kyoto University, Kyoto, Japan; 2 Department of Psychosomatic Internal Medicine, Kagoshima University Graduate School of Medical and Dental Sciences, Kagoshima, Japan; 3 Department of Nephrology, Hirakata Kohsai Hospital, Hirakata, Japan; 4 Department of Analytical and Bioinorganic Chemistry, Kyoto Pharmaceutical University, Kyoto, Japan; 5 Department of Nephrology, Graduate School of Medical Sciences, Kumamoto University, Kumamoto, Japan; 6 Institute for Clinical and Translational Science, Nara Medical University, Kashihara, Japan; 7 Division of Food Science and Biotechnology, Graduate School of Agriculture, Kyoto University, Uji, Japan; 8 Department of Nephrology, Ohkubo Hospital, Tokyo, Japan; 9 TMK Project, Medical Innovation Center, Kyoto University Graduate School of Medicine, Kyoto, Japan; 10 Research Unit/Nephrological & Endocrinological Science, Sohyaku, Innovative Research Division, Mitsubishi Tanabe Pharma Corporation, Toda, Japan; 11 Department of Diagnostic Imaging and Nuclear Medicine, Kyoto University Graduate School of Medicine, Kyoto, Japan; 12 TK Project, Medical Innovation Center, Kyoto University Graduate School of Medicine, Kyoto, Japan; 13 Department of Molecular and Clinical Pharmacology, School of Pharmaceutical Sciences, University of Shizuoka, Shizuoka, Japan; 14 Department of Nephrology and Kidney Research, Shizuoka General Hospital, Shizuoka, Japan

## Abstract

Neutrophil gelatinase-associated lipocalin (NGAL, lipocalin 2 or LCN2) is an iron carrier protein whose circulating level is increased by kidney injury, bacterial infection and obesity, but its metabolic consequence remains elusive. To study physiological role of LCN2 in energy homeostasis, we challenged female *Lcn2* knockout (KO) and wild-type (WT) mice with high fat diet (HFD) or cold exposure. Under normal diet, physical constitutions of *Lcn2* KO and WT mice were indistinguishable. During HFD treatment, *Lcn2* KO mice exhibited larger brown adipose tissues (BAT), consumed more oxygen, ate more food and gained less body weights as compared to WT mice. When exposed to 4 °C, KO mice showed higher body temperature and more intense ^18^F-fluorodeoxyglucose uptake in BAT, which were cancelled by β3 adrenergic receptor blocker or iron-loaded (but not iron-free) LCN2 administration. These findings suggest that circulating LCN2 possesses obesity-promoting and anti-thermogenic effects through inhibition of BAT activity in an iron-dependent manner.

## Introduction

Body weight is controlled by a balance between food intake and energy expenditure^1,2^. White adipose tissue (WAT) plays a key role in energy storage, whereas brown adipose tissue (BAT) is important for thermogenesis and maintenance of body temperature^1,3^. It has been recently recognized that not only rodents but also humans possess BAT^4,5^. Central nervous system controls BAT activity by sympathetic nerves through β adrenergic receptors expressed on the surface of BAT cells^1,6,7^. Therefore, strategies to activate β adrenergic receptors have been intensively screened in a hope to identify new treatment modality against obesity^2,8^.

Neutrophil gelatinase-associated lipocalin (NGAL, lipocalin 2, LCN2 or siderocalin) belongs to the lipocalin superfamily, which is a group of globular carrier proteins for hydrophobic ligands^9,10^. LCN2 carries siderophores, which are organic compounds secreted by microorganisms or plants as iron chelators for iron acquisition from their environment^11,12^. We have reported that serum LCN2 levels in patients receiving hemodialysis are positively and tightly correlated to nutritional status^13^. Consistently, Wang *et al*. reported that serum LCN2 levels are positively correlated to body mass index, a marker of obesity, in participants of a cardiovascular risk study^14^. Retinol binding protein 4, as well as LCN2, belongs to the same superfamily and is an adipokine secreted from WAT, modulating insulin signaling^15^. There is a hot and unsolved controversy as to a role of LCN2 in high fat diet (HFD)-induced obesity and insulin resistance^16–18^. Guo *et al*. reported that *Lcn2* knockout (KO) mice develop more severe obesity when fed with HFD compared to wild-type (WT) mice^16^. To the striking contrary, Law *et al*. showed that *Lcn2* KO mice exhibit less obese phenotypes than WT mice after HFD treatment^17^.

In the present study, using *Lcn2* knockout (KO) and WT mice with intervention of HFD treatment or cold exposure, we investigated a role and mechanism of LCN2 in energy homeostasis of mice.

## Results

### HFD increased blood LCN2 levels and *Lcn2* mRNA expression in WAT of WT mice

**Figure 1:**
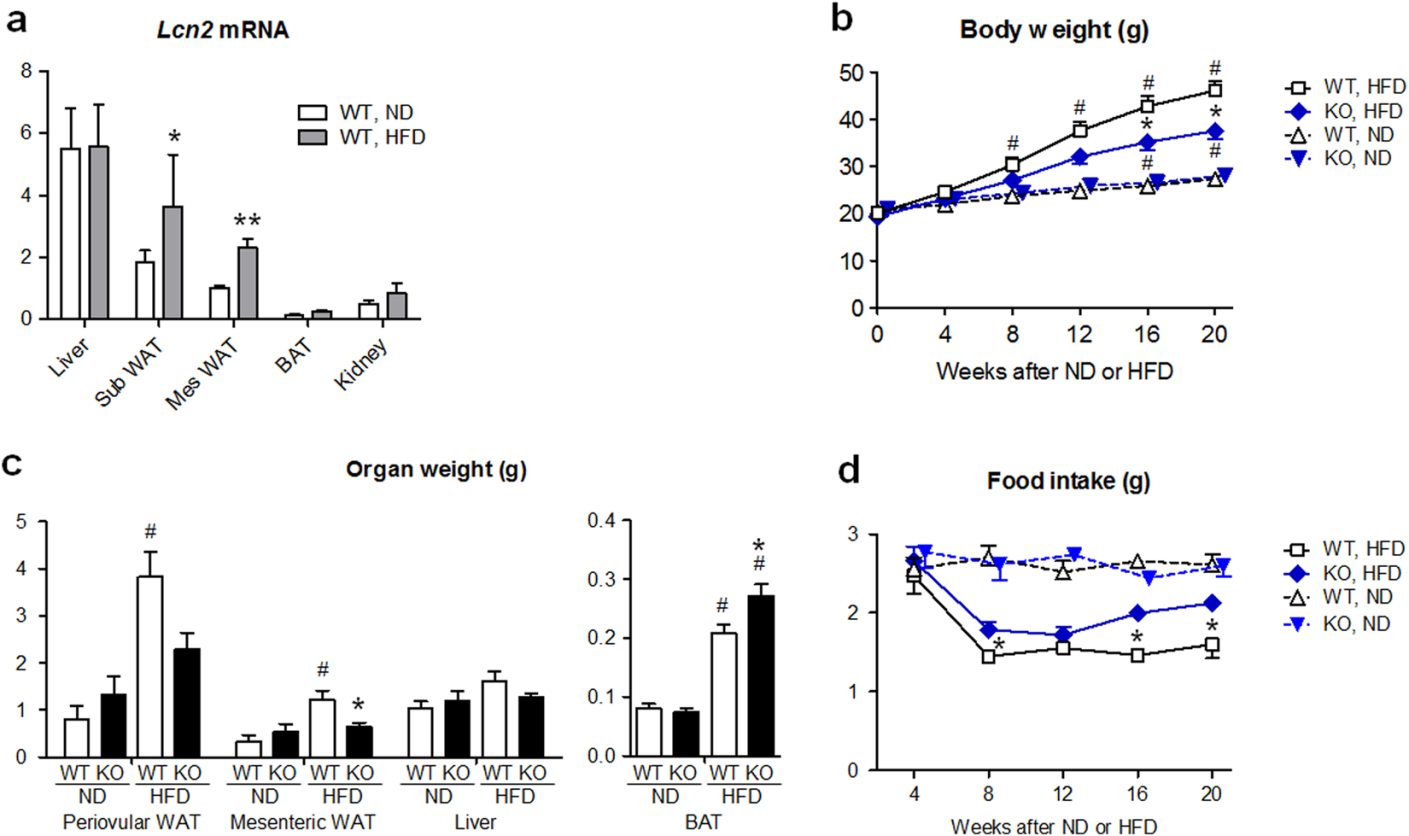
*Lcn2* KO mice were resistant against diet-induced obesity. (**a**) *Lcn2* mRNA expression levels in liver, subcutaneous (sub) WAT, mesenteric (mes) WAT and interscapular BAT at 24 weeks after HFD or ND feeding in WT mice (n = 4). Allocation to HFD or ND was carried out at 8 weeks of age. *Lcn2* expression level was normalized for 18 S ribosomal RNA expression. The level in Mes WAT given ND was defined as 1.0. *P < 0.05, **P < 0.01 vs ND. (**b**) Body weights of KO and WT mice treated with ND or HFD since 8 weeks of age (n = 9). *P < 0.05 between KO and WT mice. ^#^P < 0.05 vs 0 week. (**c**) Organ weights of KO and WT mice treated with ND or HFD for 24 weeks (n = 5–6). *P < 0.05 between KO and WT mice. *P < 0.05 vs WT. ^#^P < 0.01 vs ND. (**d**) Daily food intake of KO and WT mice (n = 6). *P < 0.05 between KO and WT mice. Data are expressed as mean ± SEM.

First, we examined the effects of HFD (60% of energy as fat, for 24 weeks during 8-32 weeks of age) upon *Lcn2* mRNA expression in the liver and adipose tissues of WT female mice (Fig. 1a). After 24 weeks, HFD increased *Lcn2* gene expression levels in the mesenteric (2.3-fold, P < 0.01) and subcutaneous fat (2.0-fold, P < 0.05) compared to normal diet (ND, 10% of energy as fat). *Lcn2* expression in the liver was not different between HFD and ND treated mice, and that in BAT was much lower than the liver and WAT. Serum LCN2 levels were 1.5-fold higher in HFD- compared to ND-treated mice at 20 weeks of treatment (185 ± 19 vs. 124 ± 15 ng/ml, n = 5, P < 0.05), as previously described^19^.

### *Lcn2* KO mice were resistant against obesity and insulin resistance induced by HFD

Body weights were similar between *Lcn2* KO and WT mice fed ND (Fig. 1b). After 20 weeks of HFD feeding, body weights of WT mice were 1.7-fold higher than ND feeding (P < 0.01). In KO mice, body weight gain by HFD was markedly reduced compared to WT mice (P < 0.05). Difference in body weights of KO and WT mice after HFD was not obvious in male mice (Supplementary Fig. S1), and further analysis was carried out. When given ND, the periovular and mesenteric WAT and the interscapular BAT weights were similar between KO and WT mice (Fig. 1c). After HFD feeding, the periovular and mesenteric WAT weights in WT mice were 4-fold larger (P < 0.001, respectively) compared to ND, and mesenteric WAT weights in KO mice were significantly smaller compared to WT mice (P < 0.05), reflecting body weight changes in KO and WT mice. On the other hand, BAT weights in KO mice given HFD were 31% larger compared to WT mice (P < 0.05). The amount of food intake (in g/day) was similar among *Lcn2* KO and WT mice during ND feeding (Fig. 1d). When fed on HFD, the amount of food intake was significantly larger in KO mice (P < 0.05). These findings indicate that KO mice under HFD consumed more food but gained less body weights compared to WT mice.

**Figure 2:**
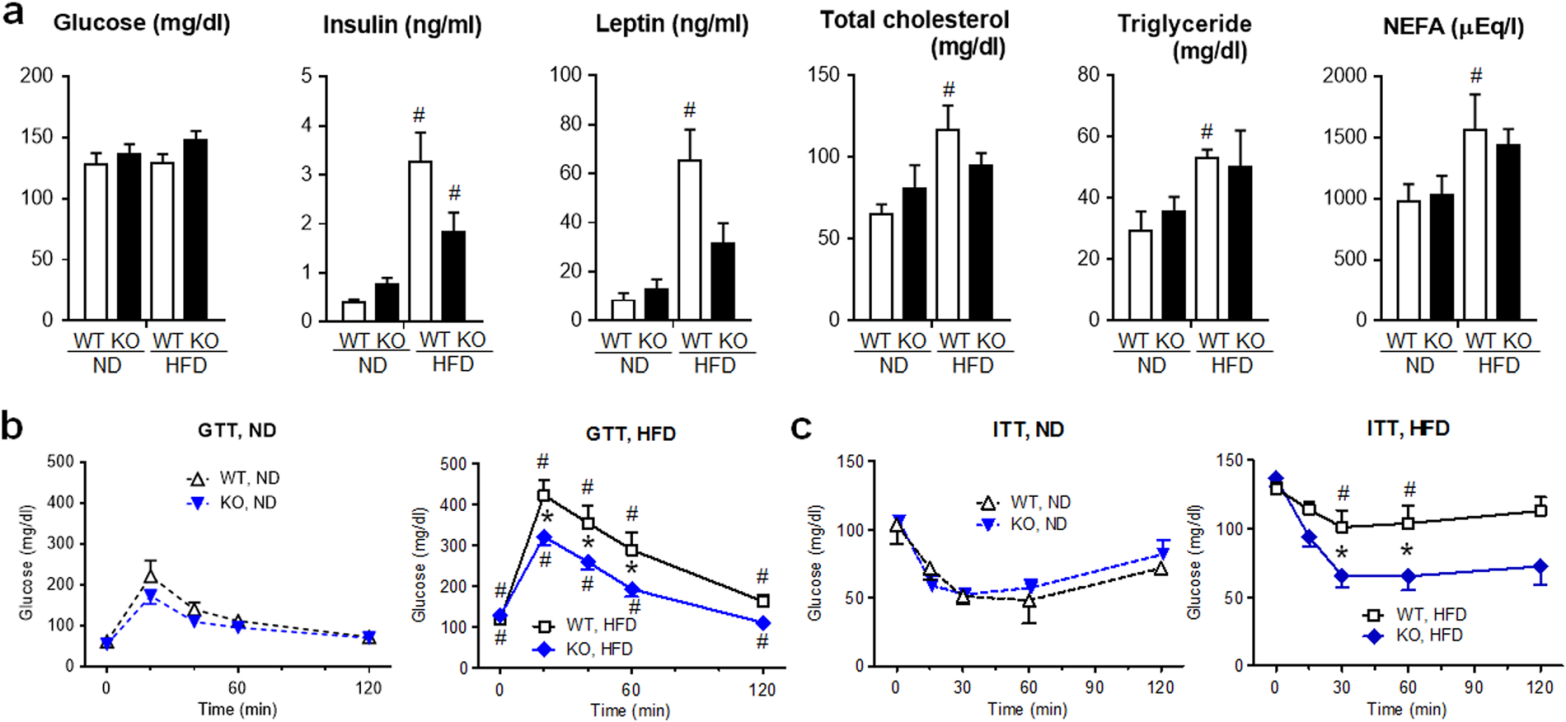
*Lcn2* KO mice developed less insulin resistance and tended to exhibit lower levels of blood metabolic parameters after HFD treatment. (**a**) Blood levels of ad libitum-fed, glucose, insulin, leptin, total cholesterol, triglyceride and non-esterified fatty acid NEFA) after 24 weeks of HFD or ND treatment in KO and WT mice (n = 5). (**b**) Glucose tolerance test (GTT). After 24 weeks of HFD or ND treatment, glucose (1 g/kg body weight) was injected intraperitoneally after 12 h fasting, and glucose levels in the blood from tail vein were serially monitored (n = 5). (**c**) Insulin tolerance test (ITT). Regular insulin (0.3 U/kg body weight for ND or 0.4 U/kg body weight for HFD) was injected intraperitoneally after 6 h fasting (n = 5). ^#^P < 0.05 vs ND. *P < 0.05 between KO and WT mice.

There was no difference in basal blood glucose levels between *Lcn2* KO and WT mice given either ND or HFD (Fig. 2a). Concerning metabolic parameters after HFD, blood insulin, leptin, total cholesterol, triglyceride and non-esterified fatty acid (NEFA) levels of WT mice were significantly elevated than those of WT mice fed on ND (P < 0.05, respectively). In KO mice given HFD, these levels tended to be lower than those in WT mice.

To compare insulin sensitivity between genotypes, we performed glucose tolerance test (GTT) and insulin tolerance test (ITT). There was no significant difference in changes of blood glucose levels after glucose or insulin injection between ND-treated KO and WT mice (Fig. 2b,c). WT mice given HFD showed significantly elevated blood glucose levels at 0 to 120 min (P < 0.05) after glucose injection compared to WT mice given ND. When HFD-treated mice were compared, KO mice showed significantly lower blood glucose levels at 20 to 60 min than WT mice (P < 0.05). In ITT, HFD-treated WT mice showed significantly higher blood glucose levels at 30 and 60 min (P < 0.05) compared to ND-treated WT mice. When we compared insulin-injected, HFD-treated mice, KO mice showed significantly lower blood glucose levels at 30 and 60 min than WT mice (P < 0.05). These findings indicate that KO mice developed less insulin resistance after HFD treatment than WT mice.

### After HFD treatment, *Lcn2* KO mice exhibited increased mRNA expression of thermogenic genes in BAT and increased oxygen consumption compared to WT mice

**Figure 3:**
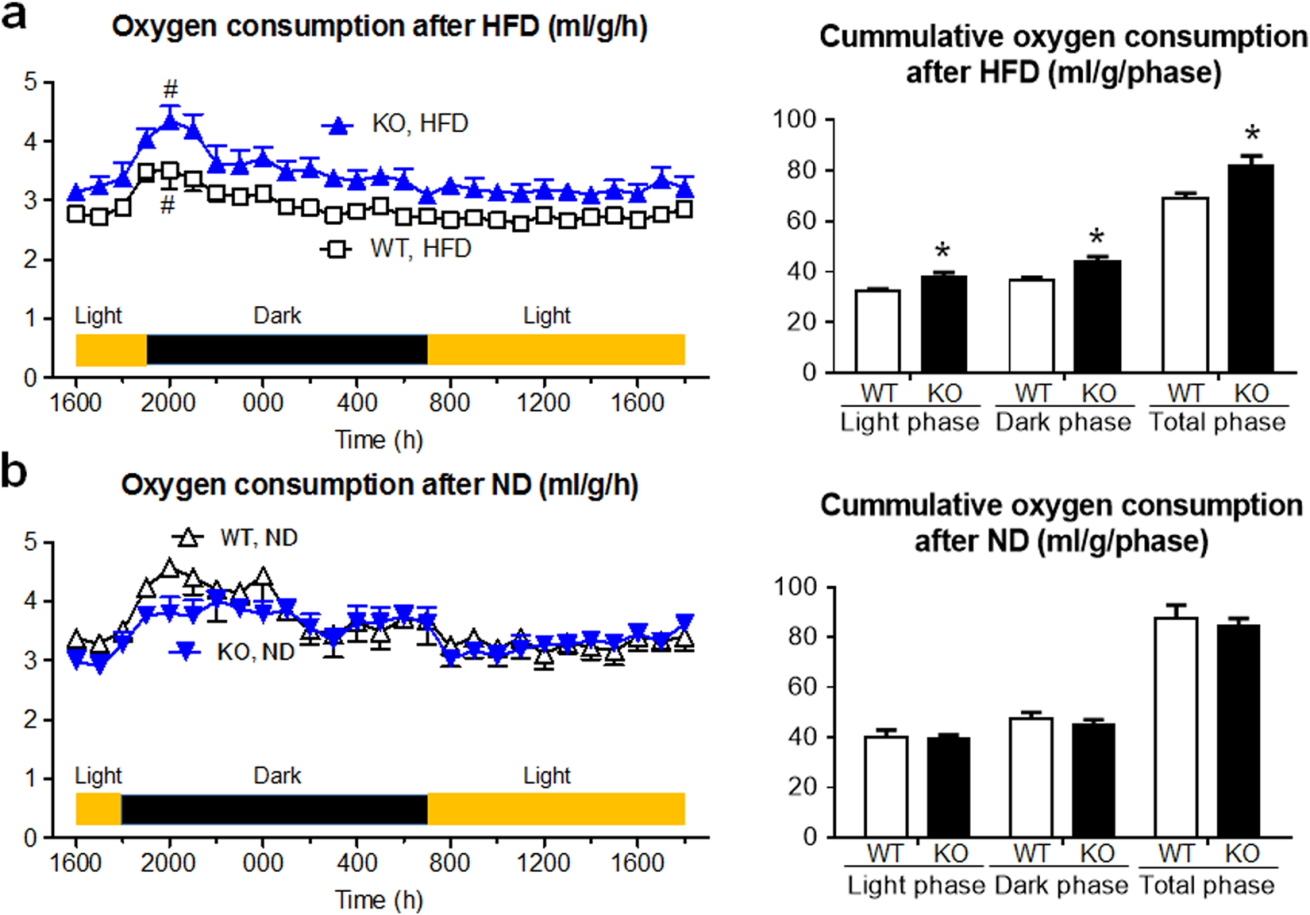
Oxygen consumption was increased in *Lcn2* KO mice after HFD. After 24 weeks of (**a**) HFD or (**b**) ND treatment, (left) hourly and (right) cumulative oxygen consumption was examined (n = 4). Marked surge was observed in HFD-fed KO and WT mice between 1800 and 2000 p.m. (^#^P < 0.05). *P < 0.05 between KO and WT mice.

Findings so far show that *Lcn2* KO mice were resistant against diet-induced obesity, and BAT activity might be more enhanced in KO than WT mice after HFD. As an indicator of BAT activity, we examined oxygen consumption (Fig. 3a,b). There was no difference in oxygen consumption between *Lcn2* KO and WT mice fed on ND. On the other hand, under HFD, oxygen consumption of KO mice was significantly larger compared with WT, both in the light and dark phases. There was no difference in locomotor activity between KO and WT mice fed both on ND or HFD (Supplementary Fig. S2).

**Figure 4:**
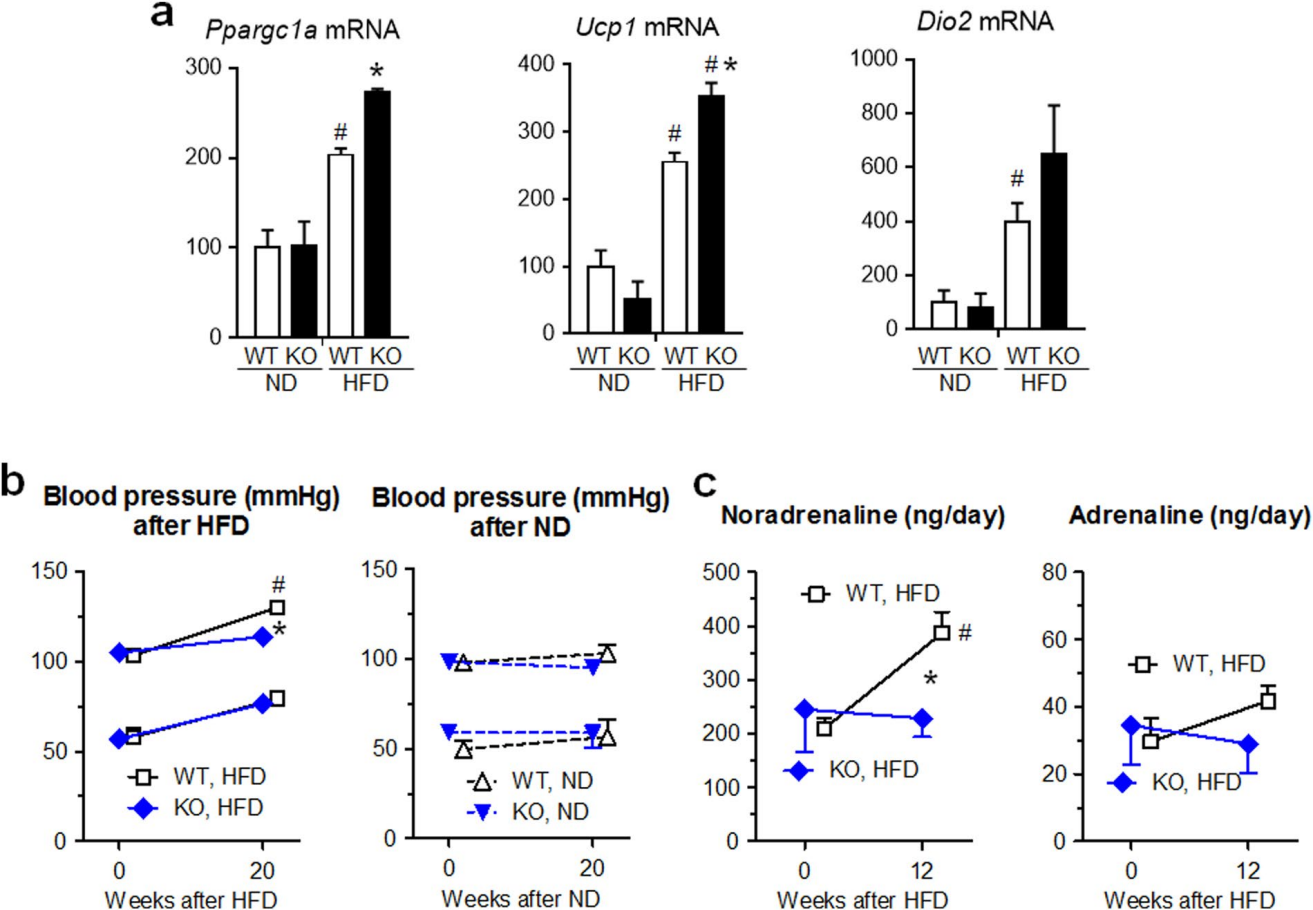
Thermogenic genes were activated in BAT, but neither blood pressure nor urinary noradrenaline level was elevated by HFD in *Lcn2* KO mice. (**a**) *Ppargc1a*, *Ucp1* and *Dio2* mRNA expression levels in interscapular BAT at 24 weeks after HFD or ND feeding in KO and WT mice (n = 5). The level in WT mice given ND was defined as 100(%). ^#^P < 0.05 vs ND. *P < 0.05 between KO and WT mice. (**b**) Systolic and diastolic blood pressures of KO and WT mice treated with HFD or ND (n = 6). (**c**) Daily urinary excretion of noradrenalin and adrenalin (n = 4). ^#^P < 0.05 vs 0 week. *P < 0.05 between KO and WT mice.

Next, we examined gene expression of key regulators of thermogenic activity in BAT (Fig. 4a), which are peroxisome proliferator-activated receptor gamma coactivator 1α (*Ppargc1a*), uncoupling protein 1 (*Ucp1*)^20^ and type 2 iodothyronine deiodinase (*Dio2*)^21^. In WT BAT, *Ppargc1a*, *Ucp1* ^1^ ^22^, and *Dio2* mRNA levels were significantly increased by HFD compared to ND (P < 0.05, respectively). In KO mice fed on HFD, *Ppargc1a* and *Ucp1* mRNA levels, which are controlled by sympathetic nerve, were further increased compared to those in WT mice with HFD (P < 0.05, respectively), suggesting that sympathetic nerve might be more activated in KO mice than WT mice under HFD.

We further studied gene expression of beige marker *Ucp1* in WAT after HFD treatment (Supplementary Fig. S3). *Ucp1* expression pattern in periovular WAT was very similar to that in BAT (Fig. 4a) and was the highest in HFD-treated *Lcn2* KO group.

Systolic blood pressure and urinary noradrenaline levels in WT mice were elevated by HFD treatment (P < 0.05), as reported previously in mice with sympathetic nerve activation^23^, but not in KO mice (Fig. 4b,c). These findings suggest that systemic sympathetic nerve activity was not activated in KO mice fed with HFD, and is consistent to less obese phenotypes in KO compared to WT mice treated with HFD. However, there is still a possibility that thermogenic, sympathetic nerve/BAT axis may be activated locally in HFD-treated KO mice.

### Obesity-inhibitory effects of *Lcn2* gene disruption was cancelled by transgenic overexpression of *Lcn2* in the liver

**Figure 5:**
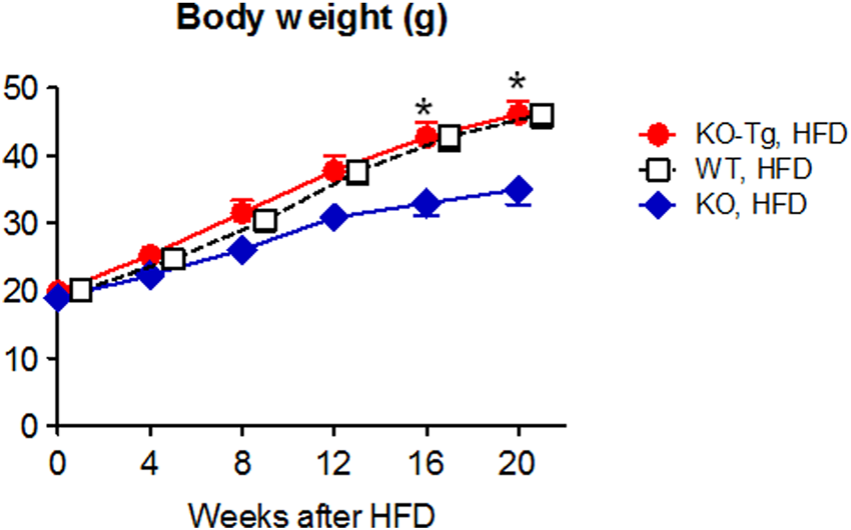
Transgenic overexpression of *Lcn2* gene in liver canceled lean phenotype of HFD-treated *Lcn2* KO mice. *P < 0.05 between KO-Tg and KO mice (n = 7).

As shown above, *Lcn2* mRNA expression level was relatively low in BAT (Fig. 1a). On the other hand, KO mice fed on HFD exhibited enlargement of BAT (Fig. 1c), upregulation of thermogenic gene expression in BAT (Fig. 4a) and increased oxygen consumption compared to WT mice with HFD (Fig. 3a). These findings imply that LCN2 may exert its actions non-cell-autonomously: for instance, in an endocrine manner. To investigate this possibility, we generated a liver-specific *Lcn2* transgenic (Tg) mouse line, in which *Lcn2* gene was overexpressed under control of serum amyloid P component (SAP) promoter. At 28 weeks of age, *Lcn2* Tg mice exhibited 5-fold higher circulating LCN2 protein levels (615 ± 90 ng/ml, n = 5, P < 0.01) compared to WT mice under ND. Gene expression of *Lcn2* in BAT was not altered in *Lcn2* Tg mice (data not shown). We mated *Lcn2* Tg mice with *Lcn2* KO mice under C57BL/6 J genetic background and observed that obesity resistance in *Lcn2* KO mice under HFD was completely rescued in *Lcn2* KO-Tg mice (Fig. 5), suggesting that activities of LCN2 protein upon energy homeostasis and metabolism were exerted in an endocrine manner^24^.

### During cold exposure, *Lcn2* KO mice showed higher body temperature and larger ^18^F-fluorodeoxyglucose uptake in BAT

**Figure 6:**
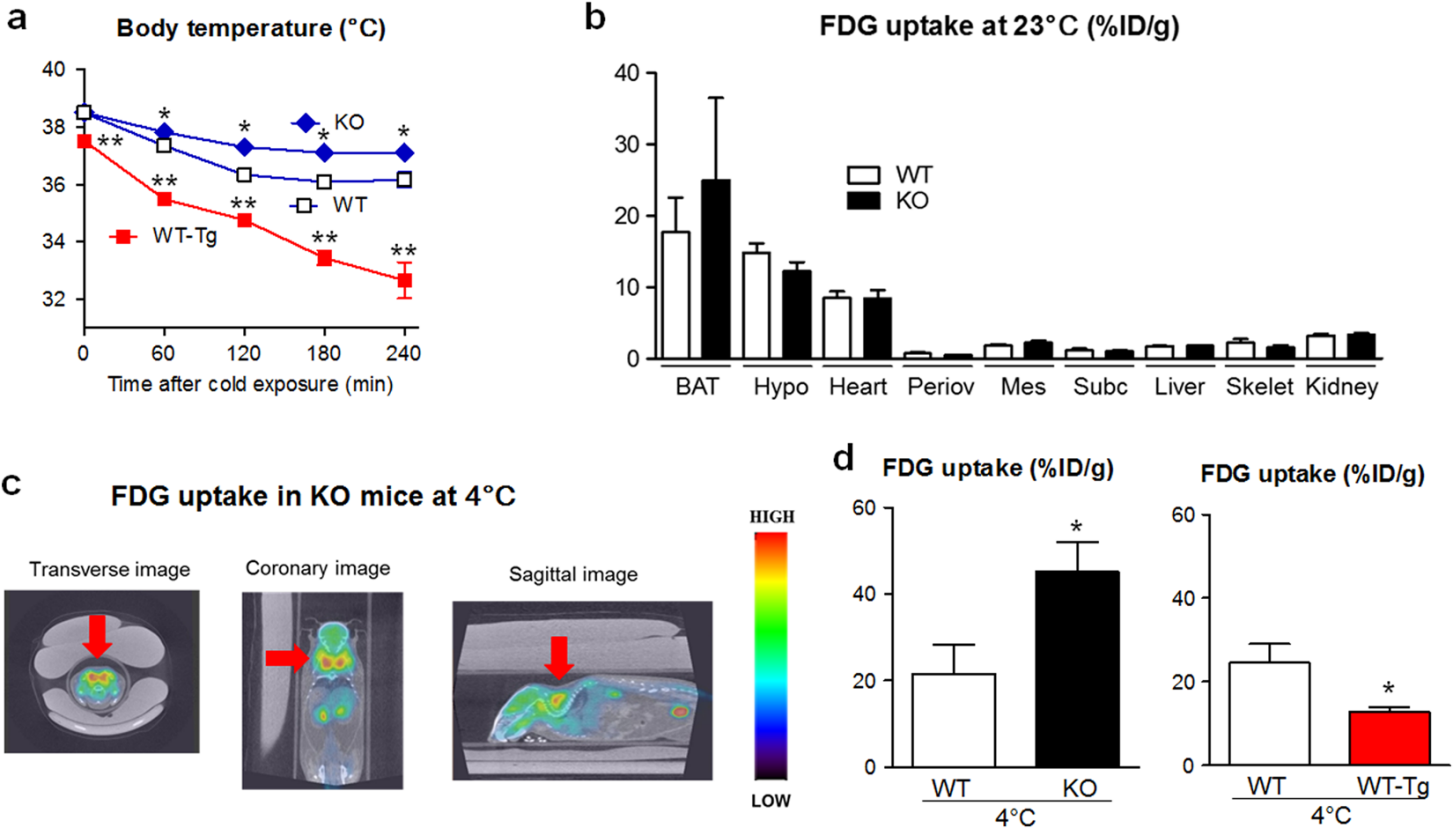
*Lcn2* KO mice were resistant against cold-induced body temperature reduction. (**a**) Change of core body temperatures among KO, WT and WT-Tg animals during 4 °C exposure (n = 6–8). *P < 0.05 or **P < 0.01 vs WT mice. (**b**) Tissue distribution of ^18^F-FDG uptake in KO and WT mice after 60 min at ambient temperature (n = 5–6). BAT, interscapular BAT; Hypo, hypothalamus; Periov, periovular WAT; Mes, mesenteric WAT; Subc, subcutaneous WAT; Skelet, skeletal muscle. (**c**) ^18^F-FDG accumulation in organs of KO mice after 60 min of cold exposure by PET-CT. Red arrow, interscapular BAT. (**d**) ^18^F-FDG uptake in BAT, calculated as % injected dose/g body weight (%ID/g), was enhanced in KO and repressed in WT-Tg mice compared to WT mice after 60 min cold exposure (n = 5–8). *P < 0.05 vs WT mice.

To study a role of LCN2 protein further in the regulation of BAT activity, we treated *Lcn2* KO and WT mice with cold exposure. Exposure of WT and KO mice to 4 °C environment resulted in gradual decrease of core body temperature, but KO mice showed significantly higher body temperature at 60–240 min after treatment (P < 0.05, Fig. 6a). After 4 h of cold exposure, *Lcn2* gene expression levels in organs including subcutaneous and mesenteric WAT were slightly elevated but did not reach statistical significance (Supplementary Fig. S4). Serum LCN2 levels were elevated by 1.5-fold after 4 h of cold exposure (202 ± 28 vs. 132 ± 11 ng/ml, n = 5, P < 0.05). On the other hand, *Lcn2* WT-Tg mice exhibited markedly lower body temperature compared to WT mice during cold exposure at 0-240 min (P < 0.01).

To quantitate functional activity of BAT during cold exposure, we injected^18^F-labelled fluorodeoxyglucose (FDG), a glucose analogue, to KO and WT mice^1,20^.^18^F-FDG uptake was prominent in interscapular BAT, hypothalamus and heart at 23 °C ambient temperature at similar levels between KO and WT mice (Fig. 6b). After cold exposure for 60 min, interscapular BAT was by far the organ most intensely taking up^18^F-FDG in KO mice, surpassing the activity of brain by positron emission tomography-computer tomography (PET-CT, Fig. 6c). ^18^F-FDG uptake in BAT of 60 min cold exposure-treated *Lcn2* KO mice was 2.1-fold larger than WT mice (P < 0.05, Fig. 6d). By contrast, *Lcn2* WT-Tg mice showed 50% less uptake than WT mice (P < 0.05).

**Figure 7:**
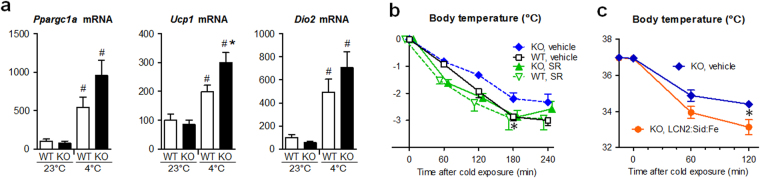
Cold tolerance in *Lcn2* KO mice was canceled by β3 adrenergic receptor antagonist or by iron-loaded LCN2. (**a**) Thermogenic gene expression in BAT was activated by cold exposure in KO and WT mice (n = 4–5). ^#^P < 0.05 vs 23 °C. (**b**) Effects of β3 adrenergic receptor antagonist SR59230A (SR) upon cold tolerance in KO mice (n = 4). *P < 0.05 between KO:vehicle and WT: vehicle mice. (**c**) Effects of LCN2:siderophore:iron complex upon cold tolerance in KO mice (n = 8). The complex was injected intraperitoneally 15 min before cold exposure. *P < 0.05.

Next, we examined gene expression in BAT of cold-exposed mice (Fig. 7a). After 4 h of 4 °C treatment, *Ppargc1a, Ucp1* and *Dio2* mRNA levels were increased by 5.4, 2.0 and 4.9-fold in BAT of WT mice, respectively (P < 0.05). Furthermore, mRNA levels of *Ucp1* was significantly higher (P < 0.05) and *Ppargca1* and *Dio2* tended to be higher in cold-exposed KO mice than in cold-exposed WT mice. These findings suggest that BAT activity was enhanced more in KO mice than in WT mice under cold conditions.

Above mentioned findings suggest that, after cold exposure, sympathetic nerve activity or sensitivity to noradrenaline was enhanced in BAT of KO mice compared to WT mice. To examine the former possibility, we measured noradrenaline levels in BAT before and after cold exposure (Supplementary Fig. S5). BAT noradrenaline levels were similar among WT, *Lcn2* KO and WT-Tg mice at ambient temperature. Importantly, previous reports indicated that BAT noradrenaline levels are decreased by acute cold exposure due to enhanced turnover of noradrenaline^25–27^. Consistently, after 4 h of cold exposure, BAT noradrenaline levels were significantly decreased in each group of animals. Furthermore, after cold exposure, BAT noradrenaline levels were significantly lower in KO mice and significantly higher in WT-Tg mice compared to WT mice. These findings imply that sympathetic nerve activity at BAT ending might be enhanced in KO mice and suppressed in WT-Tg mice compared to WT mice after cold exposure.

### Administration of β3 adrenergic receptor blocker or iron-loaded LCN2 cancelled cold tolerance in *Lcn2* KO mice

Findings so far implicate that LCN2 protein inhibits BAT activity in an endocrine and potentially β3 adrenergic receptor-mediated manner. When *Lcn2* KO mice were pretreated with SR59230A (SR), an antagonist for β3 adrenergic receptor which is specifically expressed on the surface of BAT and WAT cells^1,2^, cold exposure resulted in larger body temperature decline and the difference between KO and WT mice became negligible, especially at 180 min after cold exposure (Fig. 7b). Moreover, intraperitoneal injection of LCN2:siderophore:iron complex lead to enhanced body temperature loss in *Lcn2* KO mice compared to vehicle injection at 120 min after cold exposure (P < 0.05, Fig. 7c). In control experiments, apo-LCN2, which was ligated with neither siderophore nor iron, did not significantly affect body temperature of KO mice during cold exposure (Supplementary Fig. S6). These findings suggest that circulating LCN2 exerted anti-thermogenic effects through inhibition of sympathetic brown adipose tissue activation, in an iron-dependent manner.

### Changes in gene expression levels of LCN2 receptors and β3 adrenergic receptor in BAT

As functional receptors for LCN2, megalin (LRP2) and brain-type organic cation transporter (BOCT or SLC22A17) have been described^28–31^. *Lrp2* mRNA level in BAT was less than 0.1% of kidney. HFD treatment for 24 weeks increased *Slc22a17* gene expression by 2.5 fold (P < 0.05) in BAT of WT mice, and cold exposure for 4 h did not alter *Slc22a17* expression (Supplemental Fig. S7a). *Slc22a17* expression levels in BAT were not different between WT and KO mice. However, these findings do not demonstrate that BAT activity inhibition by LCN2 was mainly exerted by direct action upon BAT.

HFD or cold exposure did not significantly affect β3 adrenergic receptor (*Adrb3*) mRNA expression in BAT of WT and KO mice (Supplemental Fig. S7b). *Adrb3* expression levels between WT and KO were similar.

## Discussion

In the present study, firstly we show that *Lcn2* KO female mice were resistant against diet-induced obesity compared to WT mice. HFD-treated KO mice exhibited larger BAT and upregulation of thermogenic gene expression in BAT, and consumed more oxygen than HFD-treated WT mice, suggesting that increased energy expenditure was the primary cause of phenotypes in KO mice. It appears that smaller WAT mass in KO mice given HFD lead to better insulin sensitivity compared to WT mice, and larger food intake during HFD in KO mice than in WT mice was a compensatory response against energy loss. Secondly, we report that KO mice were resistant against cold exposure as to maintenance of body temperature compared to WT mice. As an indicator of BAT activity, ^18^F-FDG uptake was enhanced in cold-exposed KO mice than in WT mice. Thirdly, transgenic overexpression of *Lcn2* exhibited multiple metabolic effects including enhancement of diet-induced obesity, reduction of body temperature during cold exposure and suppression of cold-induced ^18^F-FDG uptake. Furthermore, administration of adrenergic β3 antagonist or iron-loaded LCN2 (LCN2: siderophore:iron complex) cancelled the difference between KO and WT mice during cold exposure. These findings suggest that circulating LCN2 possesses obesity-promoting and anti-thermogenic effects through suppression of BAT activity.

The mechanism how LCN2 inhibits thermogenic activity of BAT can be divided into two possibilities: blockade of sympathetic β3 activity upon BAT and suppression of BAT sensitivity to noradrenaline (namely, presynaptic and postsynaptic mechanisms). We found difference in the abundance of BAT noradrenaline contents between KO and WT mice after cold exposure (Supplementary Fig. S5), favoring the former possibility but this is only an indirect evidence. Recently, Mosialou *et al*. showed that LCN2 crosses the blood-brain barrier and directly affects hypothalamic neurons^32^.

We used *Lcn2* KO and WT female mice in this study, since the genetic difference after HFD was not obvious in male mice (Supplementary Fig. S1). Some of the reported phenotypes in *Lcn2* KO are prominent in female mice, which may be related to estrogen^33^. Likewise, after HFD treatment, toll-like receptor 4 KO female mice develop less severe insulin resistance compared to WT female mice, but these genetic difference is not observed in males^34^. Pedersen *et al*. reported that *Ucp1* gene expression in BAT was significantly lower in ovariectomized, estrogen-deficient rats compared to either intact rats or estrogen-substituted ovariectomized rats^35^. Furthermore, Rodriguez-Cuenca *et al*. revealed that BAT of female rats is 10-times more sensitive to β3 adrenergic receptor agonist compared to males^36^. These findings may be in line with the latter possibility of LCN2 action (post synaptic mechanism).

Iron depletion to obese, diabetic KKAy mice^37^ and *Lcn2* gene disruption in HFD-induced obesity in this study exerted similar effects, leading to reduced WAT weights and improved glucose tolerance and insulin resistance by GTT and ITT. Furthermore, iron depletion reportedly caused increased oxygen consumption^38^. These findings are in line with our observation that iron repletion using LCN2:siderophore:iron complex cancelled hyper thermogenic (and energy consuming) status of BAT during cold exposure in *Lcn2* KO mice. Although brown color of BAT reflects abundant content of iron-containing mitochondria, a role of iron in BAT itself has not been characterized well so far. Gene expression of one of LCN2 receptors BOCT was detected in BAT, but this does not prove that direct target of LCN2 action was BAT.

A role of LCN2 in insulin resistance and metabolic derangement induced by HFD is highly and surprisingly controversial^16–18^. A group lead by Chen and Guo has reported almost completely opposite findings from ours concerning phenotypes of *Lcn2* KO mice after HFD treatment or cold exposure^16,39^. It is quite difficult to guess the reason for discrepancy between our work and others, but it might be possibly related to the amount of iron and siderophores (such as plant-derived polyphenols) contained in the animal food^12^, intestinal flora^40^, and the age of animals to start HFD.

As to transcriptional regulation of *Lcn2* gene, NF-kappaB and CCAAT/enhancer-binding protein (C/EBP) pathways are the major contributors^10,41–44^. These mechanisms may be involved in enhanced LCN2 production in obesity. Activation of toll-like receptor 4/NF-kappaB signaling in WAT is reported during obesity^34^. Induction of *Cebp* and *Lcn2* gene expression is associated with adipocyte maturation^19^.

One of the most important actions of LCN2 reported so far is growth suppression of several pathogen by iron chelation, such as *Escherichia coli*, *Mycobacterium tuberculosis* and *Klebsiella pneumoniae*, which use siderophores fitting well in the pocket of LCN2 protein structure^11,13,41^. In this work, transgenic overexpression of LCN2 protein alone was sufficient to obtain metabolic effects, whereas ligation with iron was necessary to exert acute effects by recombinant LCN2 protein administration in cold exposure experiments. In our previous work, siderophore was required for kidney protection from renal ischemia-reperfusion injury (rIRI) by acute LCN2 injection^29^. Although at least a portion of iron-free and siderophore-free LCN2 protein (apo-LCN2) can form a complex of LCN2:endogenous siderophore:iron complex in the circulation^12^, the process may take some time and long-term presence of LCN2 in the blood may be required to form functionally sufficient amounts of the iron complex, as the case observed in *Lcn2* transgenic mice.

A number of studies have elucidated a usefulness of blood or urine LCN2/NGAL concentrations in the early detection of acute kidney injury^10,29,45^. For the kidney, LCN2 has an effect to enhance proximal tubule proliferation^29^. This effect is beneficial in an acute model of kidney injury, such as rIRI^29^, but is detrimental in chronic model of kidney injury, such as subtotal nephrectomy^46^. If endogenously-induced or exogenously-injected LCN2 reduces body temperature during acute kidney injury^47^, it might be a new mechanism of organ protection exerted by LCN2.

In conclusion, we here propose that circulating LCN2 possesses an endocrine activity to inhibit BAT activity, which becomes obvious when BAT is activated by HFD or cold exposure.

## Methods

### Experimental animals

The numbers of animals housed in animal cages were strictly controlled in each set of experiments, since they largely affect metabolic phenotypes. To minimize interference from maternal genotypes and fetal nutritional status^48^, littermates were compared. Female *Lcn2* KO, WT, WT-Tg and KO-Tg mice with C57BL/6 J genetic background (CLEA Japan, Tokyo, Japan) were studied, except for male in Supplementary Fig. S1. Mice were housed in specified pathogen-free mouse facility in Kyoto University Graduate School of Medicine with unrestricted access to chaw (F-2, 3.8 kcal/g body weight, 12% of energy as fat; Funahashi Farms, Chiba, Japan) and water at 23 °C. At 8-weeks of age, 3 mice per cage were randomly assigned to ND (D12450, 3.9 kcal/g, 10% of energy as fat; Research Diets, New Brunswick, NJ, USA) or HFD (D12492, 5.2 kcal/g, 60% of energy as fat; Research Diets). To generate *Lcn2* Tg mice, a DNA fragment carrying SAP promoter^49,50^, murine *Lcn2* cDNA and polyA signal was microinjected into pronuclei of fertilize eggs. All animal experiments were conducted in accordance with the Guidelines for Animal Research Committee of Kyoto University Graduate School of Medicine, and were approved by the Animal Experimentation Committee of Kyoto University Graduate School of Medicine.

### Measurement of metabolic parameters

Food quantity was measured for 3 consecutive days to determine mean daily food intake. Tissue and blood collection was carried out at 32 weeks of age. Serum triglyceride, total cholesterol (Wako Pure Chemicals, Osaka, Japan) and NEFA levels (Eiken Chemicals, Tochigi, Japan) were measured using enzymatic method. Glucose level was determined using Glutest Ace (Sanwa Kagaku, Nagoya, Japan). Insulin and leptin levels were measured by enzyme-immuno-assay (Morinaga Institute of Biological Science, Yokohama, Japan). Human regular insulin (Humalin R; Novo Nordisk, Bagsvaerd, Denmark) was used for ITT. Serum LCN2 levels were determined by enzyme-linked immunosorbent assay (BioPorto Diagnostics, Hellerup, Denmark).

Blood pressure was determined as mean of 6 consecutive measurements by indirect tail-cuff method with MK-2000ST (Muromachi Kikai, Tokyo, Japan).

To extract catecholamine, BAT samples were weighed and homogenized on ice in 10 volumes of 0.4 N HClO_4_ buffer (containing 2 g/L ethylenediamine tetraacetate-2Na and 20 mg/L ascorbic acid). Supernatant was collected after centrifugation at 3500 rpm for 20 min at 4 °C. Noradrenaline and adrenaline levels in urine or BAT were determined by high-performance liquid chromatography–electrochemical detection (HLC-725CAII, Tosoh Bioscience, Tokyo)^23,51^.

### Oxygen consumption and locomotor activity

Mice were individually placed in air-tight 15 × 15 × 15 cm plexi glass cages, and oxygen consumption was measured for 24 h by indirect calorimetry with MK-5000RQ and MMS-2 software (Muromachi Kikai). Spontaneous locomotor activity was measured in SUPERMEX apparatus and Compact AMS3 software (Muromachi Kikai). Mice were acclimated to monitoring for 1 h once a day for 3 days before 24-h recording.

### Body temperature study

For cold exposure experiments, F-2 chaw-treated littermate mice at 20–23 weeks of age were individually housed in 4 °C cages and core body temperature was measured by a rectal temperature probe (BAT-7001H, Bio-research, Nagoya, Japan) every hour. Mice were acclimated to measurement by a series of 10 readings once a day for 3 days before recording. A sensor was inserted 10 mm from the anus. After cold exposure, organs were dissected and frozen at −80 °C immediately. For β3 adrenergic receptor blockade, SR (5 mg/kg body weight; Sigma, St. Louis, MO, USA) or vehicle was ip injected once a day for 3 days and at 30 min before cold exposure. For LCN2 administration experiments, *Lcn2* KO mice were ip injected with 30 μg of recombinant mouse LCN2 protein expressed in and purified from mammalian cells (R&D Systems, Minneapolis, MN, USA) preincubated with equimolar of ferric enterochelin (0.8 μg, siderophore from *E. coli*; EMC Microcollections, Tuebingen, Germany)^29^ or with LCN2 protein alone (without ferric enterochelin) dissolved in phosphate-buffered saline.

### Real-time quantitative reverse transcription (RT)-PCR

Immediately after decapitation, interscapular BAT and hypothalamus were dissected out, followed by WAT (periovular, mesenteric, subcutaneous fat), heart, liver, skeletal muscle, kidney and intestine. Tissues were immediately frozen at -80 °C until analysis. Total RNA was extracted using glass-Teflon homogenizer and RNeasy Lipid Tissue Mini Kit (QIAGEN, Hilden, Germany), according to the manufacture’s instruction, and cDNA in each sample was synthesized by High Capacity cDNA Reverse Transcription Kit (Applied Biosystems, Foster City, CA, USA). TaqMan real-time PCR was performed using Premix Ex Taq (Takara Bio, Otsu, Japan) and StepOnePlus Real Time PCR System (Applied Biosystems) as described previously^52^. Primer and probe sequences are shown in Table 1. Expression levels of all genes were normalized by 18 S ribosomal RNA levels, whose primers and probe were purchased from Applied Biosystems.

**Table 1:** Primer and probe sequences for real-time quantitative RT-PCR.

Gene	Forward primer	Reverse primer	Probe
*Lcn2*	5′-GGCAGCTTTACGATGTAGAGCA -3′	5′-TCTGATCCAGTAGCGACAGCC -3′	5′-FAM-CATCCTGGTCAGGGACCAGGACCAG -TAMRA-3′
*Ppargc1a*	5′-GGAGTCTGAAAGGGCCAAACA -3′	5′-GTCAGGTCTGATTTTACCAACGTA -3′	5′-FAM-ACACGGCGCTCTTCAATTGCTTTCTGC -TAMRA-3′
*Ucp1*	5′-AAGCGTACCAAGCTGTGCG-3′	5′-CAGGACCCGAGTCGCAGAA-3′	5′-FAM-ACACCAAGGAAGGACCGACGGCCT-TAMRA-3′
*Dio2*	5′-CTCCTAGATGCCTACAAACAGGTTA-3′	5′-TCAGCGGTCTTCTCCGAGG-3′	5′-FAM-AAGATGCTCCCAATTCCAGTGTGGTGCA-TAMRA-3′
*Lrp2*	5′-TGCCCAAGCTGCCAAGCT-3′	5′-CACACCGATGTCCATGTTCACA-3′	5′-FAM-AGCCCCTGATCTGAAAGTCACCCCGT-TAMRA-3′
*Slc22a17*	5′-TGGGGCTGTGGGATTATCTGA-3′	5′-CAGAAGCAAGGAGGGTACTGAG-3′	5′-FAM-TGCTGCCATCACAACCTTCTCTGTCCT-TAMRA-3′
*Adrb3*	5′-CCTGAAACAAGCGGGTGTCTC-3′	5′-CAGCAGCTCCTTCCTGGCA-3′	5′-FAM-ACCTGAGGCAACCCCTCTGTCCCTGT-TAMRA-3′

### ^18^F-FDG uptake

^18^F-FDG uptake study was performed in Radioisotope Research Center of Kyoto University as described previously^53,54^. After 6 h fasting, saline containing 9.25 MBq of^18^F-FDG was injected through tail vein. Mice were individually housed in 23 °C or 4 °C cages for one hour and scanned by Triumph (TriFoil Imaging Inc., Chatsworth, CA, USA) under 2.5% constant isoflurane anesthesia (with 3.5 l/min of oxygen) using an acquisition time of 5 min for PET, followed by CT for 10 min. After scanning, organs were collected immediately and radiological dose of each organ was measured by gamma counter (COBRA II, Packard Instrument Company, Meriden, CT, USA).

### Statistical analysis

Data are expressed as mean ± SEM. Statistical analyses were done by two-tailed Student’s t-test for comparisons of two groups. Analysis of variance and appropriate post hoc analysis were used for comparisons of more than three groups. GraphPad Prism6 was used for analysis. P < 0.05 was considered significant.

## Electronic supplementary material

Supplementary Information
