# Supplementary material for: Obesity-promoting and anti-thermogenic effects of neutrophil gelatinase-associated lipocalin in mice
Source: Sci Rep. 2017 Nov 14;7:15501. doi: 10.1038/s41598-017-15825-4 (PMC5686189; doi:10.1038/s41598-017-15825-4)
Supplement: Supplementary file 1 — Supplementary Information [file 41598_2017_15825_MOESM1_ESM.pdf]

Supplementary Figures for Ishii et al.

Obesity-promoting and anti-thermogenic effects of neutrophil gelatinase-associated lipocalin in mice

Akira Ishii[1], Goro Katsuura[2], Hirotaka Imamaki[1,3], Hiroyuki Kimura[4], Keita P. Mori[1], Takashige Kuwabara[5], Masato Kasahara[6], Hideki Yokoi[1], Kousaku Ohinata[7], Tomoko Kawanishi[8], Junichi Tsuchida[9,10], Yuji Nakamoto[11], Kazuwa Nakao[12], Motoko Yanagita[1,9], Masashi Mukoyama[5] & Kiyoshi Mori[9,13,14]

[1]Department of Nephrology, Graduate School of Medicine, Kyoto University, Kyoto, Japan.
[2]Department of Psychosomatic Internal Medicine, Kagoshima University Graduate School of Medical and Dental Sciences, Kagoshima, Japan.
[3]Department of Nephrology, Hirakata Kohsai Hospital, Hirakata, Japan.
[4]Department of Analytical and Bioinorganic Chemistry, Kyoto Pharmaceutical University, Kyoto, Japan.
[5]Department of Nephrology, Graduate School of Medical Sciences, Kumamoto University, Kumamoto, Japan.
[6]Institute for Clinical and Translational Science, Nara Medical University, Kashihara, Japan.
[7]Division of Food Science and Biotechnology, Graduate School of Agriculture, Kyoto University, Uji, Japan.
[8]Department of Nephrology, Ohkubo Hospital, Tokyo, Japan.
[9]TMK Project, Medical Innovation Center, Kyoto University Graduate School of Medicine, Kyoto, Japan.
[10]Research Unit/Nephrological & Endocrinological Science, Sohyaku, Innovative Research Division, Mitsubishi Tanabe Pharma Corporation, Toda, Japan.
[11]Department of Diagnostic Imaging and Nuclear Medicine, Kyoto University Graduate School of Medicine, Kyoto, Japan.
[12]TK Project, Medical Innovation Center, Kyoto University Graduate School of Medicine, Kyoto, Japan.
[13]Department of Molecular and Clinical Pharmacology, School of Pharmaceutical Sciences, University of Shizuoka, Shizuoka, Japan.
[14]Department of Nephrology and Kidney Research, Shizuoka General Hospital, Shizuoka, Japan.
Correspondence and requests for materials should be addressed to K.M. (kiyoshimori2001@gmail.com)

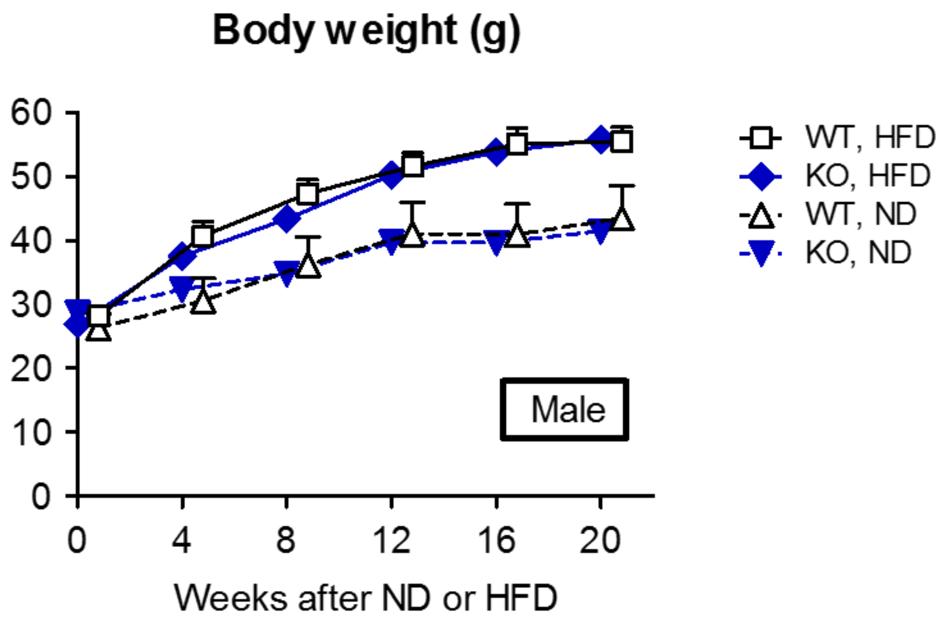

Supplementary Figure S1. Body weights of *Lcn2* KO and WT male mice treated with ND or HFD since 8 weeks of age.

Body weights were significantly elevated after 4 weeks of treatment in all 4 groups compared to 0 week, respectively ($P < 0.05$, $n = 4\text{-}8$).

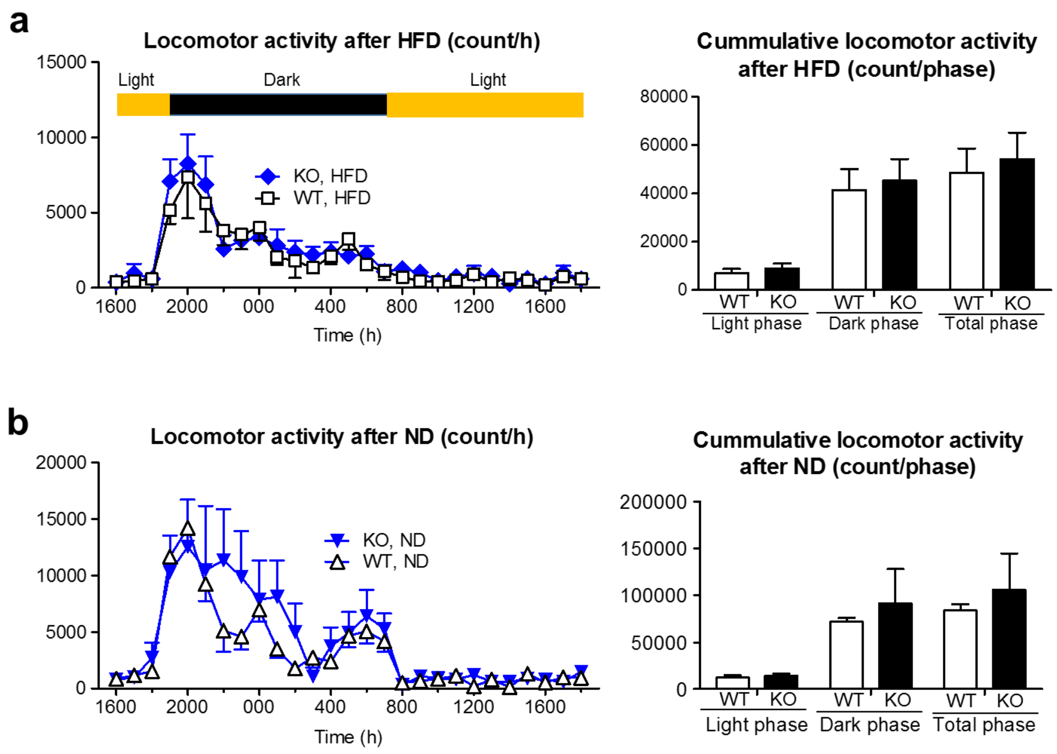

Supplementary Figure S2. Locomotor activity of *Lcn2* KO and WT female mice.

After 24 weeks of (a) HFD or (b) ND treatment, hourly (left) and cumulative (right) locomotor activities were examined (n = 4).

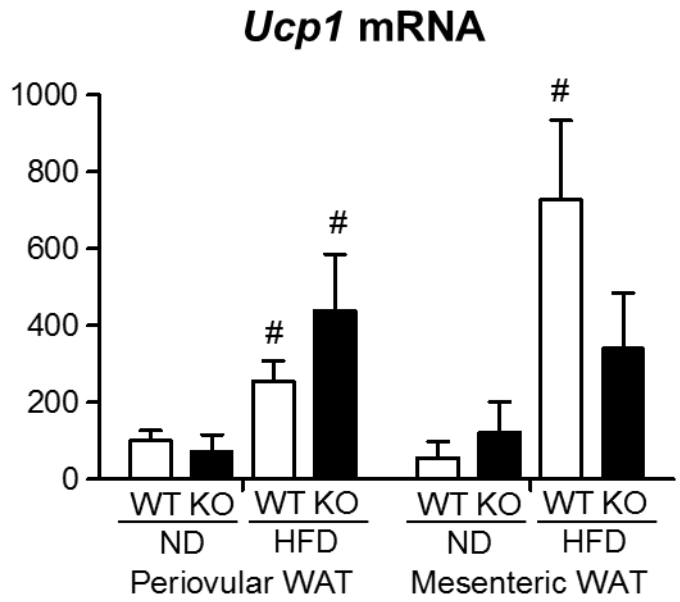

Supplementary Figure S3. Gene expression of beige marker *Ucp1* in WAT of *Lcn2* KO and WT mice after ND or HFD feeding.

After 24 weeks of HFD or ND treatment, *Ucp1* mRNA expression was examined in periovular and mesenteric WAT (n = 4-5). Gene expression level was normalized for 18S ribosomal RNA expression. The level in periovular WAT of WT mice given ND was defined as 100(%). #P < 0.05 vs ND.

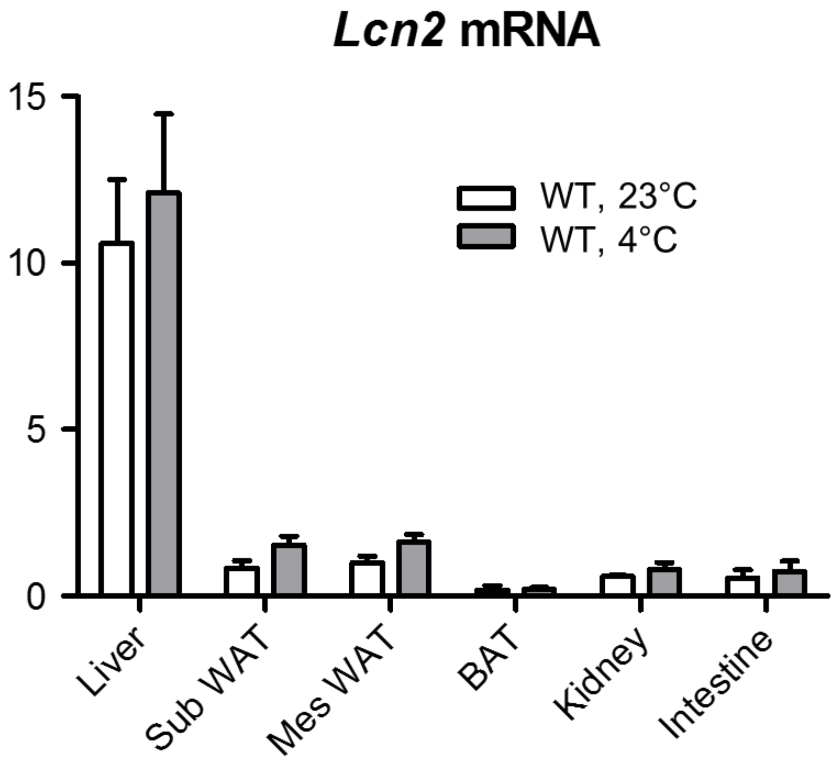

Supplementary Figure S4. *Lcn2* mRNA expression in organs of WT mice after cold exposure.

Lcn2 mRNA expression levels at 4 hours after 4°C or 23°C treatment (n = 4). The level in Mes WAT at 23°C was defined as 1.0.

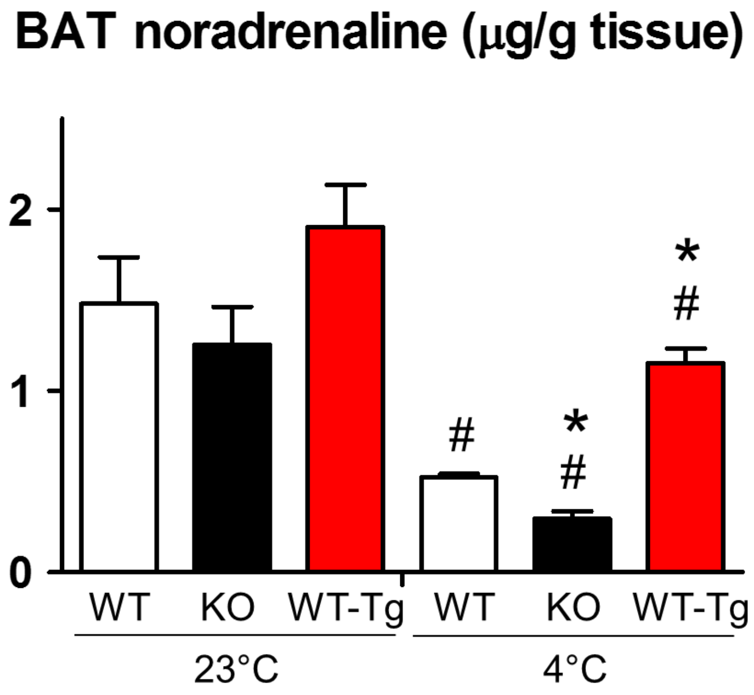

Supplementary Figure S5. Noradrenaline content in BAT after 4 h cold exposure in *Lcn2* KO, WT and WT-Tg mice (n = 5).

#P < 0.05 vs 23°C. *P < 0.05 vs WT.

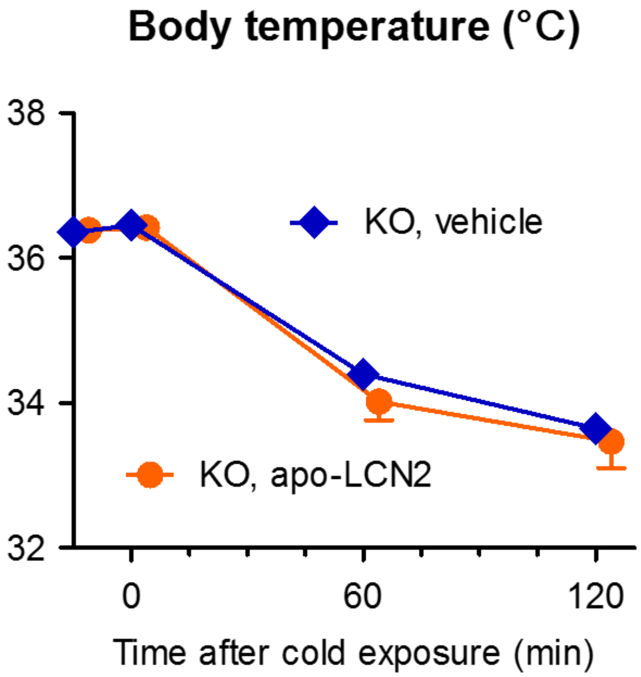

Supplementary Figure S6. Effects of apo-LCN2 (siderophore-free and iron-free LCN2) upon cold tolerance in *Lcn2* KO mice (n = 6).

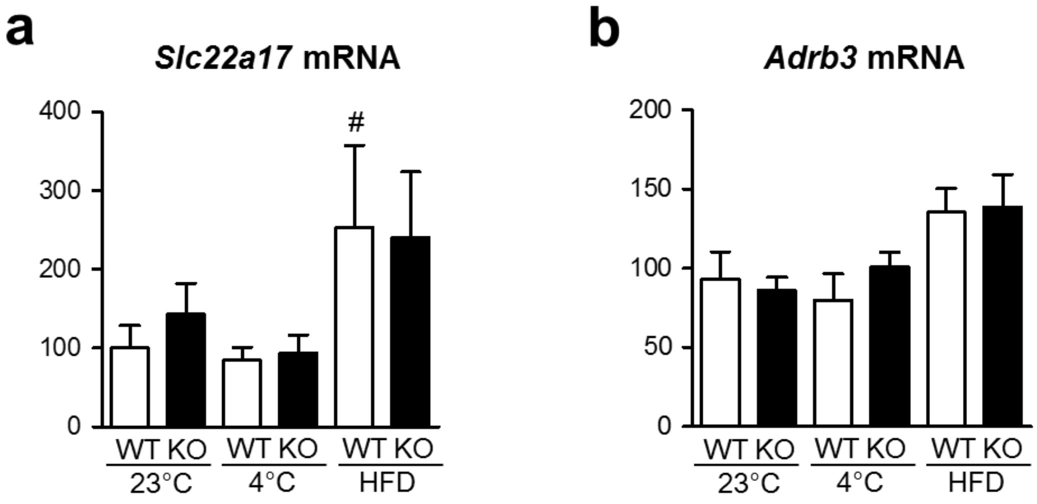

Supplementary Figure S7. *Slc22a17* and *Adrb3* mRNA expression in BAT of *Lcn2* KO and WT mice after cold exposure or HFD treatment

BAT was collected after 4 h of cold exposure or 24 weeks of HFD (n = 4-6). The level in WT BAT at 23°C given ND was defined as 100(%). #P < 0.05 vs 23°C.
